# Assessment of multi‐criteria optimization (MCO) for volumetric modulated arc therapy (VMAT) in hippocampal avoidance whole brain radiation therapy (HA‐WBRT)

**DOI:** 10.1002/acm2.12277

**Published:** 2018-02-07

**Authors:** Stephen Zieminski, Melin Khandekar, Yi Wang

**Affiliations:** ^1^ Department of Radiation Oncology Massachusetts General Hospital Harvard Medical School Boston MA USA

**Keywords:** dosimetric comparison, HA‐WBRT, MCO, treatment planning, VMAT

## Abstract

This study compared the dosimetric performance of (a) volumetric modulated arc therapy (VMAT) with standard optimization (STD) and (b) multi‐criteria optimization (MCO) to (c) intensity modulated radiation therapy (IMRT) with MCO for hippocampal avoidance whole brain radiation therapy (HA‐WBRT) in RayStation treatment planning system (TPS). Ten HA‐WBRT patients previously treated with MCO‐IMRT or MCO‐VMAT on an Elekta Infinity accelerator with Agility multileaf collimators (5‐mm leaves) were re‐planned for the other two modalities. All patients received 30 Gy in 15 fractions to the planning target volume (PTV), namely, PTV30 expanded with a 2‐mm margin from the whole brain excluding hippocampus with margin. The patients all had metastatic lesions (up to 12) of variable sizes and proximity to the hippocampus, treated with an additional 7.5 Gy from a simultaneous integrated boost (SIB) to PTV37.5. The IMRT plans used eight to eleven non‐coplanar fields, whereas the VMAT plans used two coplanar full arcs and a vertex half arc. The averaged target coverage, dose to organs‐at‐risk (OARs) and monitor unit provided by the three modalities were compared, and a Wilcoxon signed‐rank test was performed. MCO‐VMAT provided statistically significant reduction of D100 of hippocampus compared to STD‐VMAT, and Dmax of cochleas compared to MCO‐IMRT. With statistical significance, MCO‐VMAT improved V30 of PTV30 by 14.2% and 4.8%, respectively, compared to MCO‐IMRT and STD‐VMAT. It also raised D95 of PTV37.5 by 0.4 Gy compared to both MCO‐IMRT and STD‐VMAT. Improved plan quality parameters such as a decrease in overall plan Dmax and total monitor units (MU) were also observed for MCO‐VMAT. MCO‐VMAT is found to be the optimal modality for HA‐WBRT in terms of PTV coverage, OAR sparing and delivery efficiency, compared to MCO‐IMRT or STD‐VMAT.

## 1 INTRODUCTION

Brain metastases are an important source of morbidity for cancer patients. Whole brain radiation therapy (WBRT) is effective, but results in significant neurocognitive side effects for many patients, especially in terms of verbal memory. As survival for patients with metastatic brain disease increases,^1^, ^2^ approaches to spare neurocognition have become an intense area of study. Focal radiation with stereotactic radiosurgery (SRS) is one approach that results in less neurocognitive impairment,^3^ but is not an option for many patients with more diffuse metastatic disease. One alternative that has gained popularity in the last several years has been hippocampal avoidance whole brain radiation therapy (HA‐WBRT), which uses advanced radiation techniques to reduce the dose to the hippocampus, an area important for memory formation and neurogenesis.^4^ The RTOG 0933 phase II study showed evidence of improvements in quality of life and memory preservation compared to historical WBRT controls.^4^ Hopkins Verbal Learning Test‐Revised Delayed Recall (HVLT‐R) revealed a 30% mean relative decline in WBRT without hippocampal avoidance (baseline 4 months) versus 7% utilizing HA‐WBRT along with no decline in Quality of Life scores (QOL).^4^

Intensity modulated radiation therapy (IMRT) has been used as a practical delivery method for HA‐WBRT based on RTOG 0933 guidelines.^5^ Dose painting to metastatic lesions, although not required by the RTOG protocol, has also been examined.^6^ Despite these efforts, recent survey results from Slade et al.^7^ indicated 56% of radiation oncologists (*n* = 196) would not consider (IMRT) for HA‐WBRT; among several factors was the complexity of the treatment planning process which requires substantial training. More recently, volumetric modulated arc therapy (VMAT) has also been examined for HA‐WBRT.^8^ VMAT showed superior dosimetric performance to IMRT,^9^, ^10^ and can practically deliver dose painting in form of simultaneous integrated boost (SIB)^11^ to multiple brain metastases,^12^, ^13^ along with HA‐WBRT.^14^, ^15^

Dosimetric quality and efficiency for IMRT and VMAT were further promoted by a recent advancement in inverse planning technology: multi‐criteria optimization (MCO).^16^, ^17^, ^18^, ^19^, ^20^, ^21^ MCO generates a Pareto surface containing a spectrum of optimal plans, with every point on the surface representing an optimal solution with different trade‐off objectives.^16^ A user is able to navigate combinations in real‐time based on specified trade‐off objectives along with planning constraints.^16^ Numerous studies have confirmed that MCO improved plan quality over conventional inverse planning methods.^17^, ^18^, ^19^, ^20^, ^21^ In addition, MCO also reduced planning time and allowed less‐experienced treatment planners to efficiently produce high‐quality IMRT plans for complex targets in the close vicinity of numerous organs‐at‐risk (OARs), such as tumors in the head and neck region.^20^

The study aimed to compare three treatment planning methods – IMRT with MCO (MCO‐IMRT), VMAT with standard optimization (STD‐VMAT) and VMAT with MCO (MCO‐VMAT), for HA‐WBRT on complex targets with a variety of conditions (0–12 metastatic lesions with variable lesion sizes and different proximity to the hippocampus). The effectiveness of using SIB to deliver the extra dose to the metastatic lesions was also assessed for all three methods.

## 2 MATERIALS AND METHODS

### 2.A Patient selection

Ten patients previously treated with HA‐WBRT using MCO‐IMRT (eight patients in RayStation v4.0) or MCO‐VMAT (two patients in RayStation 5.0) were anonymized and re‐planned with STD‐VMAT and the other MCO modality — MCO‐VMAT in RayStation v4.7 for the eight patients originally receiving MCO‐IMRT, and MCO‐IMRT RayStation v5.0 for the two patients treated by MCO‐VMAT. The vendor (RaySearch Laboratories, Stockholm, Sweden) has not changed the IMRT optimization algorithm since RayStation v4.0, and VMAT since v4.7, which has been validated by our institutional experiences. Therefore, it was not necessary to re‐optimize all plans in the latest version.

### 2.B Computed tomography simulation

Computed tomography (CT) data were originally prepared based on the RTOG 0933 criteria. All patients had MRI with axial T2‐weighted and gadolinium contrast‐enhanced T1‐weighted sequences for hippocampus contouring with slice thickness no greater than 1.5 mm. They were immobilized in the supine position with a thermoplastic mask for a CT simulation with slice thickness of 1.25 mm with intravenous contrast. The MRI images were semi‐automatically fused to the simulation CT by an attending radiation oncologist in MIM Vista version 6 (MIM Software Inc., Cleveland, OH). Target structures (such as whole brain and distinguishable metastatic lesions), organs‐at‐risk (OARs) and external patient contour w/immobilization devices were also contoured within MIM before exporting to the treatment planning system (TPS). The hippocampus was contoured based on the RTOG contouring guidelines. The hippocampal avoidance region was generated by a 5 mm contour expansion followed by a secondary 5 mm expansion to control the dose gradient in the avoidance region. There were up to two levels of planning target volume (PTV). The hippocampal sparing whole brain PTV included the whole brain parenchyma to C1 or C2 as the clinical target volume (CTV) plus 2 mm expansion with subtraction of the hippocampal avoidance regions. The metastatic PTV was expanded from the delineated metastatic lesions with a 2 mm expansion.

### 2.C Treatment prescription

Ten patients received 30 Gy in 15 fractions to the hippocampal sparing whole brain PTV (PTV30) with an SIB of 37.5 Gy to specific metastatic PTV (PTV37.5).

### 2.D Custom optimization contours

All plans were given custom contours to guide the optimization process. These contours included PTV30−PTV37.5, a volume used to help improve dose uniformity within the whole brain region without metastatic disease. A custom target structure was created within PTV30 in between the left and right hippocampus for added control of midline target coverage. A custom avoidance structure was created inferior to the PTV30 to control excessive inferior dose to uninvolved optic regions and oral cavity.

### 2.E Treatment plan parameters

MCO‐IMRT plans utilized a step‐and‐shoot delivery method (SMLC) with eight to eleven beams at variable gantry angles (including one to three non‐coplanar angles) depending on the location of the metastatic disease. All plans were optimized using a 6‐MV photon beam on an Elekta Infinity linear accelerator employing Agility multileaf collimator (MLC, 80 pairs of 5‐mm leaves).

All VMAT plans were generated using dual coplanar full arcs (clockwise/counterclockwise of 358°, between 181° and 179°), with a fixed collimator angle (between 20° to 40°) selected based on the angle of hippocampus. A third non‐planar vertex arc swept from 1° to 179° with a 270° couch kick. All three arcs employed a control point every 2° of gantry rotation. The dose grid resolution for all plans was set to 0.2 × 0.25 × 0.2 cm^3^ (left‐right, superior‐inferior, and anterior‐posterior, respectively).

The use of multiple arcs has been shown to yield superior plan quality.^22^ Furthermore, with the dual arc feature enabled, only one set of fluence profiles are optimized while more information from the fluence maps can be kept during the leaf sequencing process. This ‘one arc’ fluence map conserves leaf motion with one arc focusing on the left side of the target and the second arc on the right side at a given control point angle, which in turn reduces the leaf openings over the OAR and increases sparing.^23^ Chen et al. reported that the use of dual arcs in VMAT resulted in notable dosimetric improvements for complex targets such as head and neck.^24^

The differences between the STD‐VMAT (a.k.a., rayArc) and MCO‐VMAT optimization in RayStation were explained by Ghandour et al.^25^ The rayArc uses a direct machine parameter optimization (DMPO) algorithm that starts with a coarse arc segmentation (24° spacing), while converting to optimized fluence maps per initial angle. The maps are then converted into a user determined 2–4 control points per initial angle, while filtering out the smallest points based on a sorting algorithm.^23^ Control points are then converted to comply with machine parameter motion constraints (e.g., max leaf speed, valid dose rates, delivery time, number of monitor units per degree) along with leaf/jaw positioning (static or dynamic). Chen et al. reported the use of small arc spacing of 2° per control point led to notable dosimetric improvements for complex targets such as head and neck.^24^

MCO empowers the user to produce a final plan by considering multiple criteria via the generation of a Pareto database followed by a navigation process which smoothly interpolates amongst the plans in the database. For a Pareto optimal plan, no criterion can be further improved without sacrificing another criterion. For photon optimization in RayStation, for a plan with n objectives defined, a minimum of 2*n* Pareto plans are needed to produce a practical approximation of the Pareto surface,^18^ whereas the use of 4n plans leads to a closer approximation to the true Pareto surface, which we use at our institute.^19^ In addition to objectives, constraints are used to focus on clinically useful plans, creating a Pareto surface with smaller range but finer resolution. At least two constraints — the minimum dose for a target and the maximum dose for an organ, are required to start the Pareto plan generation. An MCO plan is selected by navigating on the Pareto surface using the navigational sliders. A particular slider can be clamped to limit the range of navigation on the Pareto surface to prevent degradation while navigating other sliders in desired directions. Once navigated, the fluence pattern of the selected Pareto optimal plan is converted to deliverable machine parameters for each control point using a final dose calculation. In RayStation, this final deliverable plan is used for clinical evaluation.

MCO‐VMAT uses three unique algorithms: a convex Pareto surface approximation for fluence maps, a fluence map optimization for the discrete Pareto surface representation (navigational best plan) and DMPO VMAT optimization to generate MLC segments.^25^ Both VMAT optimization techniques used a single‐value pencil beam kernel decomposition for approximation to save computational time. An intermediate dose is used to minimize discrepancies between pencil beam and collapsed cone algorithm (which is used in final dose calculation).^25^

### 2.F Dosimetric and plan quality comparison analysis

#### 2.F.1 Organs‐at‐risk

With regards to hippocampal sparing, four metrics were assessed to determine plan quality: dose covering 100% volume (D100), mean dose (Dmean), point max of hippocampus (Dmax) and percent volume receiving 10 Gy (V10). Combined lens dose and cochlea dose (left and right) are assessed by point max (Dmax).

#### 2.F.2 Target coverage

The coverage of PTV30 was assessed by percentage of volume receiving 25 Gy (V25) and 30 Gy (V30), whereas the coverage of PTV37.5 and GTV37.5 was evaluated using dose covering 95% (D95) and 99% (D99) of the volume, respectively.

#### 2.F.3 Plan quality parameters

Total monitor units and Dmax (Gy) for each plan was recorded. Dose uniformity with control of the dose falloff was assessed using the volume of PTV30−PTV37.5. The V35 of PTV30 was also recorded to determine the control of dose beyond 30 Gy in the whole brain regions without metastatic disease.

#### 2.F.4 Statistical analysis

Statistical analysis was performed with a Wilcoxon signed‐rank test to determine if there was any significant difference of the parameters examined along with standard deviations (SD). A *P* value smaller than 0.05 was considered statistically significant. The comparison was conducted between MCO‐VMAT and MCO‐IMRT, as well as between MCO‐VMAT and STD‐VMAT. STD‐VMAT was not compared to MCO‐IMRT.

## 3 RESULTS

Table 1 shows the total plan MU, dose to OARs, maximum plan dose, and target coverage for the ten patients. The number of metastatic lesions, the size of the PTV and their proximity to the hippocampus are also shown along with the coverage of the metastatic PTV (PTV37.5). The results are shown for MCO‐VMAT (MV), MCO‐IMRT (MI), and STD‐VMAT (SV).

**Table 1:**
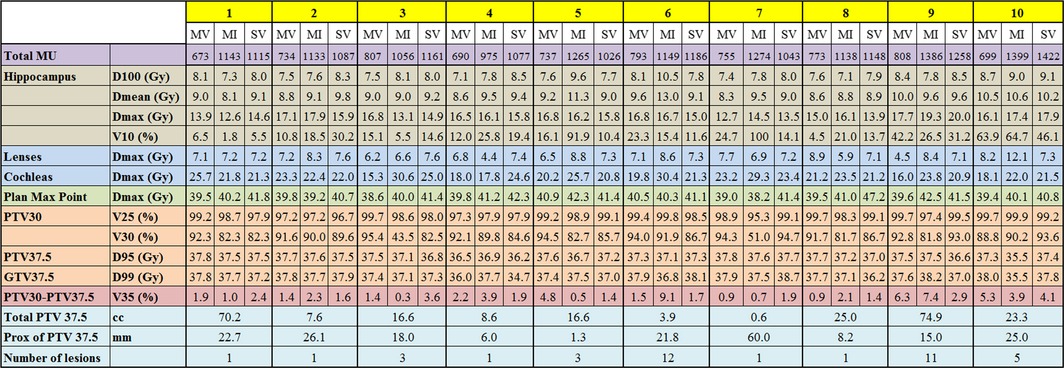
Comparison of plan performance for the ten SIB HA‐WBRT patients. MV stands for MCO‐VMAT, MI for MCO‐IMRT, and SV for STD‐VMAT

Table 2 shows the cumulative averages of the metrics illustrated in Table 1 for each treatment modality. The results for the MCO‐VMAT are compared to MCO‐IMRT and STD‐VMAT separately, to demonstrate any improvements resulted in by the use of MCO and VMAT, respectively.

**Table 2:**
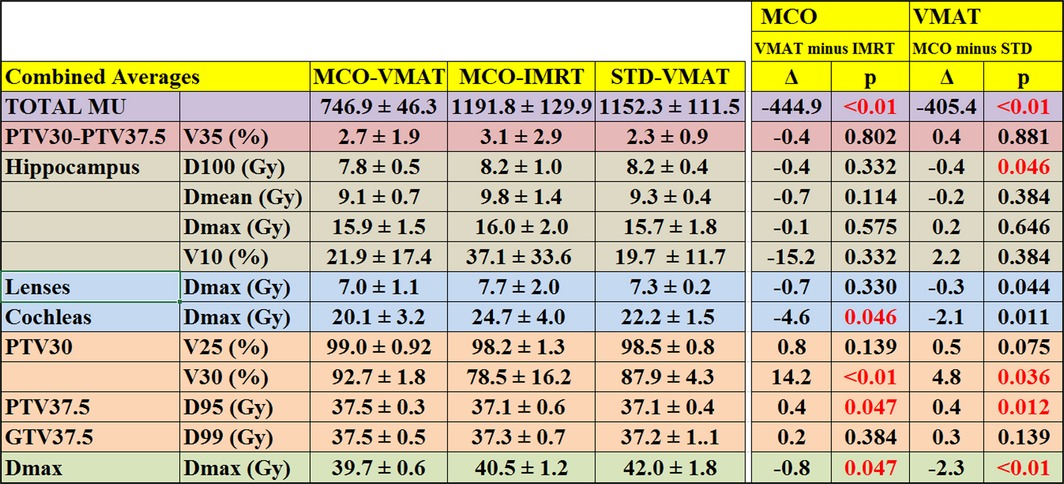
Cumulative average and standard deviation of the plan metrics shown in Table 1, for each treatment modality. The impact of using VMAT over IMRT in MCO planning is shown under the MCO column, whereas the impact of using MCO over standard optimization in VMAT planning is shown in the column of VMAT. The differences with statistical significance (*P* < 0.05) is shown in red

### 3.A OAR sparing

As shown in Table 2, the cumulative averages of D100 and Dmean for hippocampus were both lower for MCO‐VMAT compared to STD‐VMAT or MCO‐IMRT. Dmax to hippocampus was similar in all three modalities. Decrease in hippocampus D100 using MCO‐VMAT was statistically significant when compared to STD‐VMAT. Cumulative averages of the maximum dose to the lenses and that to the cochleas were lower for MCO‐VMAT, when compared to either STD‐VMAT or MCO‐IMRT.

### 3.B Target coverage

As shown in Table 2, MCO‐VMAT achieved statistically significant improvement on prescription coverage for PTV30 when compared to either MCO‐IMRT or STD‐VMAT, while maintaining lower OAR values. The standard deviation of the V30 is also smaller for MCO‐VMAT, implying improved uniformity of plan quality. The use of VMAT instead of IMRT in MCO planning resulted in an improvement of 14.2% for the V30 of PTV30, and the use of MCO for VMAT optimization led to a net gain of 4.8%. For the ten patients with metastatic disease, MCO‐VMAT provided higher D95 and D99 for PTV37.5, with the results being statistically significant for D95 difference between MCO‐IMRT and STD‐VMAT.

**Figure 1:**
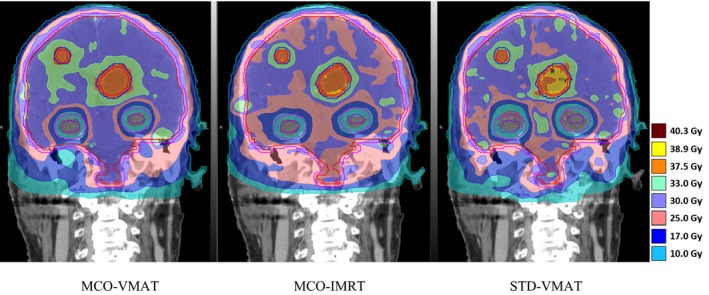
Isodose plan comparison in the coronal view for the patient that MCO‐VMAT demonstrated most improvement on the coverage of the whole brain PTV30 (patient 3 in Table 1).

Figure 1 compares the coronal view of the isodose distribution provided by the three modalities for the patient that MCO‐VMAT demonstrated most improvement on V30 of PTV30 (patient 3 in Table 1). For this patient, only 43.5% of the PTV30 was covered by the prescription dose in MCO‐IMRT, and 82.5% in STD‐VMAT. The use of MCO‐VMAT promoted the coverage to 95.4%, which is generally considered clinically desirable.

**Figure 2:**
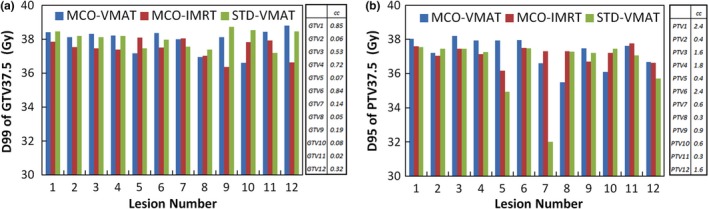
Comparison of target coverage provided by MCO‐VMAT, MCO‐IMRT and STD‐VMAT, for the patient with 12 metastatic lesions (patient 6). The results are shown for the (a) D99 of the metastatic GTV (GTV37.5) and (b) D95 of the metastatic PTV (PTV37.5) that receive the SIB. The target volumes are shown beside the charts.

**Figure 3:**
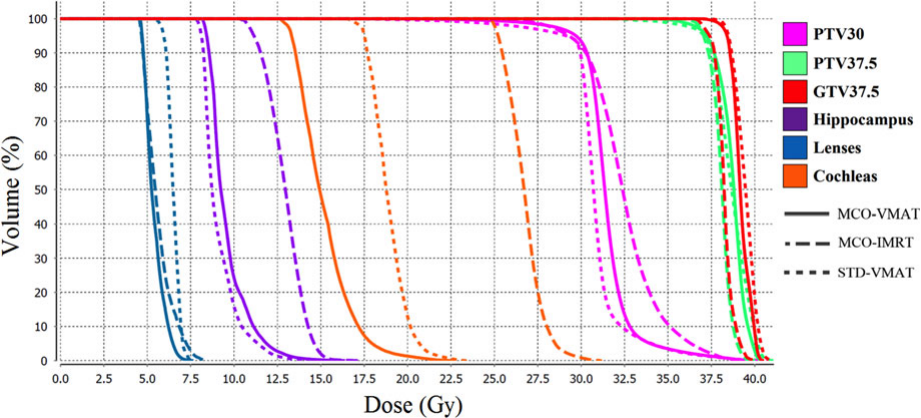
Dose volume histogram (DVH) for the patient illustrated in Fig. 2 (patient 6). The PTV37.5 and GTV37.5 are combined volumes for the 12 lesions.

Figures 2 and 3 show the dosimetric performance of the three modalities for the patient with the maximum number of metastatic lesions (12 for patient 6 as shown in Table 1). Figure 2 shows the D99 of GTV37.5 and D95 for PTV37.5. In general, the three modalities provided similar target coverage considering the challenges of multiple lesions. Figure 3 compares the dose volume histogram (DVH) for various target volumes and normal organs. Both MCO‐ and STD‐VMAT created lower hotspots in the whole brain and spared more hippocampus than MCO‐IMRT. MCO‐VMAT and MCO‐IMRT led to lower dose to the lens. MCO‐VMAT significantly reduced the dose to the combined cochleas compared to the other two modalities. Overall, MCO‐VMAT offered the best combination of target coverage and normal tissue sparing for this most challenging case.

### 3.C Treatment plan quality parameters

On average, MCO‐VMAT resulted in a lower overall plan Dmax hotspot. The decrease was 1.2 Gy and 1.8 Gy when compared to MCO‐IMRT (*P* = 0.047) or STD‐VMAT (*P* < 0.01), respectively. Dose uniformity, quantified by the volume receiving 35 Gy with the PTV30 but outside PTV37.5, observed MCO‐VMAT (2.7%), when compared to MCO‐IMRT (3.1%) or STD‐VMAT (2.3%). A significant decrease was observed for the average monitor unit (MU) for MCO‐VMAT (746.9), when compared to MCO‐IMRT (1191.8) or STD‐VMAT (1152.3). The reduction was 37% and 35%, respectively (*P* < 0.01 for both.)

## 4 DISCUSSION

All MCO‐VMAT plans (with or without the SIB to the metastatic lesions) achieved the RTOG 0933 guidelines (which only required WBRT to 30 Gy) with acceptable or better hippocampus sparing.^26^ Prior studies have highlighted MCO for its operational flexibility and planning efficiency, along with superior dosimetric performance.^21^, ^24^ With the navigation function in MCO, metrics such as dose uniformity and maximum dose can be loosened for the potential of greater OAR sparing or more target coverage. In this HA‐WBRT study, the constraints were customized based on the hippocampus volume, the number, size and location of any metastatic lesions, to allow the generation of the Pareto surface from a favorable starting position. As reported in prior studies, if the navigation is done in a very unbalanced manner or pushed towards extreme values, dose discrepancy will appear at the MLC segmentation stage when the optimizer tries to find the MLC pattern physically allowed by the linear accelerator that can mimic the fluence map achieved at the end of the navigation step.^21^ The discrepancy is also attributed to the difference in dose calculation algorithm — pencil beam in Pareto optimization and the more accurate collapsed cone in final dose calculation after the MLC segmentation. The possibility of real‐time navigation using a spectrum of Pareto‐optimal plans can lower the learning curve for planning staff and thus encourage more clinics to consider highly conformal treatment (e.g., SIB with VMAT) in very complex anatomical situations (e.g., hippocampal sparing, limbic circuit sparing).^7^, ^27^

Prokic et al. reported that the SIB technique could achieve better hippocampal sparing compared to sequential boost in form of stereotactic radiation therapy (e.g., 8 Gy × 2) after WBRT.^28^ In addition, the use of a single isocenter for the treatment of multiple brain metastasis led to reduced delivery time while maintaining the dosimetry quality, as compared to the sequential boost.^29^ Our study demonstrates that MCO‐VMAT allows for hippocampal sparing despite a variety of SIB conditions (number of lesions, size of targets and proximity to hippocampus). Also, the SIB approach allowed selective dose escalation for lesions within less than 5 mm to the hippocampus which would otherwise be rejected for protocol. As reported by Gondi et al., 3% of brain metastases in 8.6% of patients were found within 5 mm of the hippocampus (*n* = 371). 30 In our study, MCO‐VMAT achieved dose escalation to 37.5 Gy for lesions as close as 1.3 mm from the hippocampus (patient 5). This example shows the potential tradeoffs between target coverage and OAR sparing, as controlling metastatic burden is also a key factor in neurocognitive outcomes.^31^ In this case, both MCO modalities increased PTV 37.5 SIB target coverage for the tumor in close proximity to the hippocampus. However, MCO‐VMAT offered 95% coverage for PTV30, whereas MCO‐IMRT and STD‐VMAT only provided 83% and 86%, respectively, with comparable hippocampus metrics as shown in Table 1. MCO allowed user flexibility for clinicians to prioritize high‐risk target clinical objectives utilizing Pareto optimal navigational solutions. Thus, SIB with MCO‐VMAT may allow for meaningful hippocampal sparing for patients who would otherwise not be eligible due to peri‐hippocampal metastases.

## 5 CONCLUSION

MCO‐VMAT was proven superior to MCO‐IMRT or STD‐VMAT for HA‐WBRT. This study sampled patients with various numbers of metastatic lesions (0 to 12), and the lesions had a wide variety of size and location with respect to the hippocampus. On average, MCO‐VMAT improved the PTV30 coverage, with statistical significance, by 14.2% and 4.8%, respectively, compared to MCO‐IMRT and STD‐VMAT. It also slightly boosted the dose to GTV37.5 and PTV37.5. Finally, MCO‐VMAT significantly reduced the MUs, resulting in faster treatment compared to both MCO‐IMRT and STD‐VMAT.

## CONFLICT OF INTEREST

The authors declare no conflict of interest to disclose.

## References

[1] Nieder C , Spanne O , Mehta MP , Grosu AL , Geinitz H . Presentation, patterns of care and survival in patients with brain metastases: what has changed in the last 20 years? Cancer. 2011;117:2505–2512. 24048799 10.1002/cncr.25707

[2] Stelzer KJ . Epidemiology and prognosis of brain metastases. Surg Neurol Int. 2013;4:S192–S202. 23717790 10.4103/2152-7806.111296 PMC3656565

[3] Brown PD , Jaeckle K , Ballman KV , et al. Effect of radiosurgery alone vs radiosurgery with whole brain radiation therapy on cognitive function in patients with 1 to 3 brain metastases: a randomized clinical trial. JAMA. 2016;316:401–409. 27458945 10.1001/jama.2016.9839 PMC5313044

[4] Gondi V , Pugh SL , Tomé WA , et al. Preservation of memory with conformal avoidance of the hippocampal neural stem cell compartment during whole brain radiotherapy for brain metastases (RTOG 0933): a phase 2 multi‐institutional trial. J Clin Oncol. 2014;32:3810–3816. 25349290 10.1200/JCO.2014.57.2909 PMC4239303

[5] Gondi V , Tolakanashalli R , Mehta MP , et al. Hippocampal‐sparing whole brain radiotherapy: a “how‐to” technique, utilizing helical tomotherapy and LINAC‐based intensity modulated radiotherapy. Int J Radiot Oncol Biol Phys. 2010;78:1244–1252. 10.1016/j.ijrobp.2010.01.039 PMC2963699 20598457

[6] Liang X , Ni L , Hu W , et al. A planning study of simultaneous integrated boost with forward IMRT for multiple brain metastases. Med Dos. 2013;38:115–116. 10.1016/j.meddos.2012.07.010 23237662

[7] Slade AS , Stanic S . The impact of RTOG 0614 and RTOG 0933 trials in routine clinical practice: the US survey of utilization of memantine and IMRT planning for hippocampus sparing in patients receiving whole brain radiotherapy for metastases. Contemp Clin Trials. 2016;47:74–77. 26718093 10.1016/j.cct.2015.12.013

[8] Rong Y , Evans J , Xu‐welliver M , et al. Dosimetric evaluation of intensity‐modulated radiotherapy, volumetric modulated arc therapy, and helical tomotherapy for hippocampal‐avoidance whole brain radiotherapy. PLoS ONE. 2015;10:e0126222. 25894615 10.1371/journal.pone.0126222 PMC4404135

[9] Shen J , Bender E , Yaparpalvi R , et al. An efficient volumetric arc therapy treatment planning approach for hippocampal‐avoidance whole‐brain radiation therapy (HA‐WBRT). Med Dos. 2015;40:205–209. 10.1016/j.meddos.2014.11.007 25605507

[10] Lee K , Lenards N , Holson J . Whole‐brain hippocampal sparing radiation therapy: volume modulated arc therapy vs intensity‐modulated radiation therapy case study. Med Dos. 2016;46:15–21. 10.1016/j.meddos.2015.06.003 26235550

[11] Cilla S , Deodato F , Digesu C , et al. Assessing the feasibility of volumetric‐modulated arc therapy using simultaneous integrated boost (SIB‐VMAT): an analysis for complex head‐neck, high‐risk prostate and rectal cancer cases. Med Dos. 2014;39:108–116. 10.1016/j.meddos.2013.11.001 24342167

[12] Wang JZ , Pawlicki T , Rice R , et al. Intensity‐modulated radiosurgery with rapidarc for multiple brain metastases and comparison with static approach. Med Dos. 2012;37:31–36. 10.1016/j.meddos.2010.12.010 21705211

[13] Lagerwaard FJ , Van der Hoorn EA , Verbakel WF , Haasbeek CJ , Slotman BJ , Senan S . Whole brain radiotherapy with simultaneous integrated boost to multiple brain metastases using volumetric modulated arc therapy. Int J Rad Oncol Biol Phys. 2009;75:253–259. 10.1016/j.ijrobp.2009.03.029 19577856

[14] Hsu F , Carolan H , Nichol A , et al. Whole brain radiotherapy with hippocampal avoidance and simultaneous integrated boost for 1‐3 brain metastases: a feasibility study using volumetric modulated arc therapy. Int J Radiat Oncol. 2010;76:1480–1485. 10.1016/j.ijrobp.2009.03.032 19625140

[15] Gutiérrez AN , Westerly DC , Tomé WA , et al. Whole brain radiotherapy with hippocampal avoidance and simultaneously integrated brain metastases boost: a planning study. Int J Radiat Oncol Biol Phys. 2007;69:589–597. 17869672 10.1016/j.ijrobp.2007.05.038 PMC2350212

[16] Craft DL , Halabi TF , Shih HA , Bortfeld TR . Approximating convex pareto surfaces in multiobjective radiotherapy planning. Med Phys. 2006;33:3399–3407. 17022236 10.1118/1.2335486

[17] Craft DL , Hong TS , Shih H , Bortfeld TR . Improved planning time and plan quality through multicriteria optimization for intensity‐modulated radiotherapy. Int J Radiat Oncol Biol Phys. 2012;82:e83–e90. 21300448 10.1016/j.ijrobp.2010.12.007 PMC3202037

[18] Craft D , Halabi T , Shih HA , Bortfeld T . An approach for practical multiobjective IMRT treatment planning. Int J Radiat Oncol Biol Phys. 2007;69:1600–1607. 17920782 10.1016/j.ijrobp.2007.08.019

[19] Wala J , Craft D , Paly J , Zietman A , Efstathiou J . Maximizing dosimetric benefits of IMRT in the treatment of localized prostate cancer through multicriteria optimization planning. Med Dos. 2013;38:298–303. 10.1016/j.meddos.2013.02.012 23540492

[20] Kierkels RGJ , Visser R , Biijl HP , et al. Multicriteria optimization enables less experienced planners to efficiently produce high quality treatment plans in head and neck cancer radiotherapy. Biol Med Cent. 2015;10:87. 10.1186/s13014-015-0385-9 PMC4397887 25885444

[21] McGarry C , Bokrantz R , O'Sullivan JM , Hounsell AR . Advantages and limitations of navigation‐based multicriteria optimization (MCO) for localized prostate IMRT planning. Med Dos. 2014;39:205–211. 10.1016/j.meddos.2014.02.002 24630909

[22] Tang G , Earl MA , Luan S , Wang C , Mohiuddin MM , Yu CX . Comparing radiation treatments using intensity‐modulated beams, multiple arcs, and single arcs. Int J Radiat Oncol Phys. 2010;76:1554–1562. 10.1016/j.ijrobp.2009.04.003 PMC2846542 20338482

[23] RaySearch Laboratories AB . VMAT Optimization in Raystation. http://www.raysearchlabs.com/globalassets/about-overview/mediacenter/wp-re-ev-n-pdfs/white-papers/white-paper-3-vmat-aug-2015.pdf, Accessed May 1, 2016

[24] Chen H , Craft DL , Gierga DP . Multicriteria optimization informed VMAT planning. Med Dos. 2014;39:64–73. 10.1016/j.meddos.2013.10.001 PMC3954571 24360919

[25] Ghandour S , Matizinger O , Pachoud M . Volumetric‐modulated arc therapy planning using multicriteria optimization for localized prostate cancer. J Appl Clin Med Phys. 2015;16:258–269. 10.1120/jacmp.v16i3.5410 PMC5690115 26103500

[26] A phase II trial of hippocampal avoidance during whole brain radiotherapy for metastases: RTOG 0933; version date: 12/5/2011.

[27] Marsh JC , Gielda BT , Herskovic AM , Wendt JA , Turian JV . Sparing of the hippocampus and limbic circuit during whole brain radiation therapy: a dosimetric study using helical tomotherapy. J Med Imag Radiat Oncol. 2010;54:375–382. 10.1111/j.1754-9485.2010.02184.x 20718919

[28] Prokic V , Wiedenmann N , Fels F , Schmucker M , Nieder C , Grosu AL . Whole brain irradiation with hippocampal sparing and dose escalation on multiple brain metastases: a planning study on treatment concepts. Int J Radiat Oncol Biol Phys. 2013;85:264–270. 22516808 10.1016/j.ijrobp.2012.02.036

[29] Ozer A , Giem J , Yound J , Ali I , Ahmad S , Hossain S . Comparison of doses received by the hippocampus in patients treated with single isocenter – vs multiple isocenter – based stereotactic radiation therapy to the brain for multiple brain metastases. Med Dos. 2015;40:214–317. 10.1016/j.meddos.2015.04.001 25962907

[30] Gondi V , Tomé W , Marsh J , et al. Estimated risk of perihippocampal disease progression after hippocampal avoidance during whole‐brain radiotherapy: safety profile for RTOG 0933. Radiother Oncol. 2010;95:327–331. 20392503 10.1016/j.radonc.2010.02.030 PMC2981132

[31] Li J , Bentzen SM , Renschler M , Mehta MP . Regression after whole‐brain radiation therapy for brain metastases correlates with survival and improved neurocognitive function. J Clin Oncol. 2007;25:1260–1266. 17401015 10.1200/JCO.2006.09.2536

